# Investigation of the transability of dietary small non-coding RNAs to animals

**DOI:** 10.3389/fgene.2022.933709

**Published:** 2022-08-30

**Authors:** Milad Norouzi, Mohammad Reza Bakhtiarizadeh, Abdolreza Salehi

**Affiliations:** Department of Animal and Poultry Science, College of Aburaihan, University of Tehran, Tehran, Iran

**Keywords:** microRNA, xenomiR, cross-species communications, gene regulation, non-coding RNAs

## Abstract

Our daily diet not only provides essential nutrients needed for survival and growth but also supplies bioactive ingredients to promote health and prevent disease. Recent studies have shown that exogenous microRNAs (miRNAs), xenomiRs, may enter the consumer’s body through dietary intake and regulate gene expression. This fascinating phenomenon suggests that xenomiRs can act as a new class of bioactive substances associated with mammalian systems. In contrast, several studies have failed to detect xenomiRs in consumers and reported that the observed diet-derived miRNAs in the previous studies can be related to the false positive effects of experiments. This discrepancy can be attributed to the potential artifacts related to the process of experiments, small sample size, and inefficient bioinformatics pipeline. Since this hypothesis is not generally accepted yet, more studies are required. Here, a stringent and reliable bioinformatics pipeline was used to analyze 133 miRNA sequencing data from seven different studies to investigate this phenomenon. Generally, our results do not support the transfer of diet-derived miRNAs into the animal/human tissues in every situation. Briefly, xenomiRs were absent from most samples, and also, their expressions were very low in the samples where they were present, which is unlikely to be sufficient to regulate cell transcripts. Furthermore, this study showed that the possibility of miRNAs being absorbed through animals’ diets and thus influencing gene expression during specific periods of biological development is not inconceivable. In this context, our results were in agreement with the theory of the transfer of small RNAs under certain conditions and periods as xenomiRs were found in colostrum which may modulate infants’ immune systems *via* post-transcriptional regulation. These findings provide evidence for the selective absorption of diet-derived small RNAs, which need to be investigated in future studies to shed light on the mechanisms underlying the transference of diet-derived miRNAs.

## Introduction

Small RNA-mediated RNA interference (RNAi), one of the most influential discoveries in biology, is a conserved regulatory mechanism in eukaryotic cells to regulate/silence the expression of genes in a sequence-specific manner (Tufekci et al., 2004; Carthew, 2006). In this regard, small interfering RNAs (siRNAs) and microRNAs (miRNAs) are two important types of small RNAs. siRNAs are small molecules ∼21 bp (ranging between 20 and 22 bp) (Hammond, 2015) that are processed from long double-stranded RNA (dsRNAs) precursors, either transcribed from the genome (such as transposons) (Rodriguez et al., 2004), aberrant transcription products, and complementary transcripts (Ventura et al., 2008; Poliseno et al., 2011)) or exogenous RNA sequences (such as transgenes and viral replication intermediates). On the other hand, miRNAs, typically 22 bp in length, are evolutionarily endogenous regulators that are transcribed from miRNA-encoding genes [either encoded within exons/introns of genes or autonomous miRNA genes (Ventura et al., 2008; Poliseno et al., 2011)] *via* RNA polymerase II or III (Schanen and Li, 2011). Mature miRNAs are derived from longer hairpin precursors through cleavage by Drosha and Dicer enzymes and then loaded to Argonaute family proteins to facilitate the binding of miRNAs to their mRNA targets and induce RNA silencing (Achkar et al., 2016). miRNAs have been found to regulate gene expression at the post-transcriptional level through mRNA cleavage or translational repression, thus participating in a wide range of biological processes in the cell including development (Abbott et al., 2005), cell differentiation (Onnis et al., 2012), proliferation (Brennecke et al., 2003; Rodriguez et al., 2016), apoptosis (Thompson and Cohen, 2006), and metabolism (Boehm and Slack, 2006).

High-throughput sequencing-based technologies along with recent advances in bioinformatics methods have considerably expanded our insights into the different aspects of molecular biology such as miRNAs. Using these approaches, in 2012, Zhang et al. (2012) provided the first evidence of the potential roles of miRNAs in cross-species communications, called exogenous miRNAs (xenomiRs). This hypothesis suggests that exogenous miRNAs (such as miR-168a from rice in Zhang et al. study) may have the ability to be transmitted from diet to mammals through the gastrointestinal epithelial cells and regulate their endogenous targets (such as low-density lipoprotein receptor adapter protein 1, LDLRAP1, in Zhang et al. study), similar to endogenous miRNAs. After Zhang’s study, this hypothesis has received great attention, and several follow-up studies have been conducted. Some researchers found xenomiRs in the blood and tissues of animals and humans (Liang et al., 2015; Ma et al., 2017; Stephen et al., 2020), whereas others attributed diet-derived miRNAs to experimental or technical errors. For example, in agreement with Zhang et al., the results of Li et al. (2015) study showed that small food-derived RNAs can be transferred to the mammalian placenta and directly regulate fetal gene expression. In another study, the authors reported that MIR2911, derived from the honeysuckle plant, was observed in animals drinking or gavage feeding honeysuckle. In addition, the authors showed that the miRNAs were able to regulate the influenza virus by reducing replication and rescuing viral infection (Zhou et al., 2015). In contrast, several studies have failed to detect xenomiRs in various animal/human body fluids (Witwer et al., 2013), or the levels of the target xenomiRs were not higher than the background. In a comprehensive study, Kang et al. reanalyzed 824 human public datasets to detect xenomiRs in the serum, plasma, exosomes, cerebrospinal fluid, liver, and blood cells of rats. They claimed that xenomiRs showed low abundance compared to endogenous miRNAs, and potentially they originate from technical artifacts.

Despite these controversies, the xenomiR hypothesis is not generally accepted yet. One of the most important challenges to investigating this hypothesis is the lack of reproducibility of the published results as in 2012, Zhang et al. showed that rice-derived miRNAs exist in humans and mice, and their results were rejected 1 year later (Dickinson et al., 2013). Differentiation of exogenous miRNAs that share subtle (or no) differences with their homologs in host species can be challenging and confounded by inherent problems including the potential artifacts introduced in the process of experiments, small sample size, and incomplete used bioinformatics pipeline with a lack of rigorous statistical analysis (Snow et al., 2013; Witwer et al., 2013; Huang et al., 2017; Huang et al., 2018). In this regard, the applied bioinformatics pipeline is most important and affects the final results, whereas with the same dataset, one study reports a low abundance of the plant-derived miRNAs in host species, and the other study claims that the diet-derived miRNAs are very abundant. Hence, it can be most appropriate to use the same stringent and reliable bioinformatics pipeline to analyze the different datasets to investigate whether or not miRNAs from the diet can be transferred to animals/humans. Here, to address these analytical challenges, a stringent and reliable bioinformatics pipeline, including a serial cascade of computational filters, was designed, and a large number of public miRNA-Seq data obtained from different experiments were reanalyzed to detect the diet-derived miRNAs within the samples and investigate the xenomiR hypothesis.

## Materials and methods

### Data collection

A total of seven miRNA-Seq datasets related to various species and tissues, including 133 samples, were obtained from the NCBI SRA and GEO databases. It is worth noting that six out of the seven datasets were obtained from the studies that their authors did not investigate the xenomiR hypothesis, but their experimental designs enabled us to use them in the present study. In other words, these six studies were used for the first time in this study to investigate the xenomiR hypothesis. Summary of all the used studies is presented in Table 1 (for details of these studies, see Supplementary Table S1).

**TABLE 1:** Summary of the used studies in the present study.

Study number	GSE ID	Organism	Sequencing platform	Tissue	Number of samples	Overall design
1	GSE136806	*Bos taurus*	Illumina HiSeq 2000	Serum extracellular vesicle (EV)	6	Control cows were fed TMR, while treated cows were fed a diet where alfalfa hay was partly replaced with whole cotton seed and soybean hull for 30 days
2	GSE117441	*Bos taurus*	Illumina HiSeq 2000	Rumen epithelium, duodenum epithelium, jejunum epithelium, liver, and mammary gland	90	Three different diet groups (alfalfa hay, corn stover, and rice straw-based diets) were used (six dairy cows per group)
3	GSE81616	*Bos taurus*	Illumina HiSeq 2000	Mammary glands	4	Four Holstein cows at the peak of lactation received a low forage diet supplemented or not with 4% of sunflower oil
4	GSE113598	*Mus musculus*	Illumina NextSeq 500	Liver	6	Two groups (including three mice per group) were fed a normal diet and a normal diet with soy (25% soy)
5	GSE81619	*Ovis aries*	Illumina Genome Analyzer II	Hypothalamus	2	Two groups, containing 18 sheep per group, were fed a normal alfalfa diet (1.5 kg for each sheep) and alfalfa with concentrate.
6	GSE61025	*Capra hircus*	Illumina HiSeq 2500	Mammary glands	10	Ten goats were fed a hay-based diet. For 48 h before slaughtering, four goats consumed this diet *ad libitum* (Control), and the six others were food-deprived (FD).
7	GSE92897	*Sus scrofa* and *Rattus norvegicus*	Illumina HiSeq 2000 (both)	Serum (both)	15	**Rat**: Rats were divided into three groups (three animals in each group) fed two treatments of potato and rice, and the control group was fed a diet without two treatments
						**Pig:** Six pigs are divided into two groups. The first group was fed a diet based on cow’s milk, and the second group was fed a diet based on corn

### Data preprocessing

The SRA format data sets were converted to FASTQ files using fastq-dump from the SRA Toolkit (v 2.3.5) (Leinonen et al., 2011). Quality control of the raw data was assessed by FastQC (version 0.10.1) (Brown et al., 2017). Trimmomatic (version 0.36) (Bolger et al., 2014) was applied for adapter trimming and removing low-quality bases/reads using the PHRED score cut off ≥20 and read length cut off of a minimum of 18 bases. The clean reads were rechecked using FastQC to ensure sufficient quality for downstream analysis.

### Removal of the species-related RNAs

To exclude the clean reads related to non-miRNAs or miRNAs of the host, the following processes were applied, which were previously described in Gebert et al. (2017). These processes enabled us to identify the clean reads that are potentially related to exogenous RNAs, such as diet-derived miRNAs. Low complexity reads were filtered based on an advanced version of the clustering algorithm from NGS TOOLBOX (Rosenkranz et al., 2015), which has been developed in Unitas software (version 1.7.0).

Two groups of miRNA mature/hairpin sequences were downloaded from the miRBase (Kozomara and Griffiths-Jones, 2014) database (release 22) for each species: 1) miRNAs related to the species of interest (which consume the diet); 2) miRNAs related to closest species to the species of interest. Then, the clean reads were aligned against them using the SeqMap (Jiang and Wong, 2008) tool to exclude the mapped reads. The closest species (Supplementary Table S2) helped us identify the homologue miRNAs that are not annotated in the species of interest, considering the fact that most of miRNA sequences are evolutionary conserved.

For each species, sequences of rRNA from Silva (Quast et al., 2013), tRNAs from GtRNAdb (Chan and Lowe, 2016), small nuclear RNAs (snRNA) and small nucleolar RNAs (snoRNA) from Rfam (Griffiths-Jones et al., 2003), repeat sequences from Repbase (Jurka et al., 2005), and cDNAs from ENSEMBL databases were retrieved to build a reference dataset which contained a mixture of host sequences. The remaining clean reads were discarded if they were mapped to these databases, and the unmapped reads were kept for further analysis. Also, for some studies (study number two, rumen-epithelium samples) that were suspected to be contaminated with possible microbiome sequences, the reads were aligned to annotated sequences in the NCBI UniVec VecScreen database, and the mapped reads were filtered out. Aligning the reads against the mentioned databases/sequences was carried out using the Bowtie tool (v1.1.2) with two mismatches allowed.

Since the remaining clean reads may still contain RNAs related to the species of interest, such as species-specific miRNAs that are not yet annotated in the databases, they were aligned against the reference host genome (Table 2). This step makes the pipeline more conservative to identify the exogenous sequences. To perform this, Bowtie software (v1.1.2) was applied allowing up to two mismatches. The genome sequences were downloaded from the ENSEMBL database. Finally, the unmapped reads were considered potential exogenous RNAs for further analysis.

**TABLE 2:** List of the investigated species in the present study and their corresponding genome versions.

Organism	Genome version
*Bos taurus*	ARS-UCD1.2 (GCA_002263795.2)
*Mus musculus*	GRCm38.p6 (GCA_000001635.8)
*Ovis aries*	Oar_rambouillet_v1.0 (GCA_002742125.1)
*Capra hircus*	ARS1 (GCA_001704415.1)
*Homo sapiens*	GRCh38.p13 (GCA_000001405.28)
*Sus scrofa*	Sscrofa11.1 (GCA_000003025.6)
*Rattus norvegicus*	Rnor_6.0 (GCA_000001895.4)

### Exogenous miRNA identification

In this step, the possibility of the existence of xenomiRs in the samples was investigated. To do this, the remaining reads were aligned against the potential diet-derived miRNA sequences by Bowtie software (up to two mismatches). The potential diet-derived miRNA sequences were selected based on the consumed diet for each sample. To avoid false positives, as far as possible, the miRNAs with less than nine reads in at least two samples were filtered out. Finally, the expression of each potential diet-derived miRNA was quantified based on its read counts. The diet-derived hairpin miRNA sequences were obtained from the miRBase (Kozomara and Griffiths-Jones, 2014) database (release 22).

### Statistical analysis

To identify the differentially expressed diet-derived miRNAs, between treated and control groups, the DEseq2 (Love et al., 2014) package in R software (3.6) was used.

## Results

### miRNA-seq datasets

**FIGURE 1:**
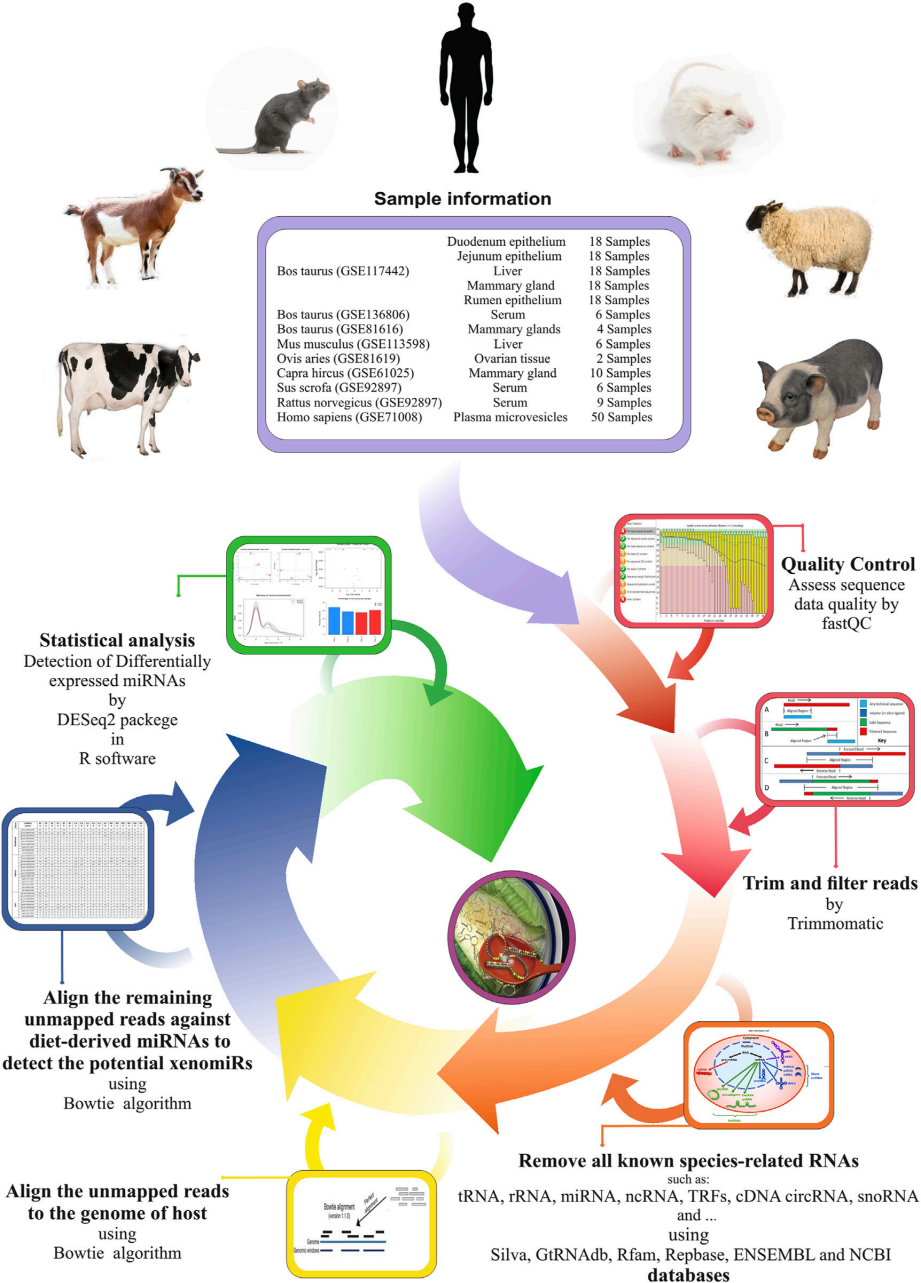
Used pipeline for endogenous and exogenous miRNA discovery to evaluate xenomiR hypothesis. The clean reads were first screened against endogenous sequence databases (host) including Silva, GtRNAdb, Rfam, Repbase, ENSEMBL, and NCBI UniVec VecScreen followed by host genomic sequence. Then, unmapped reads were aligned against diet-derived miRNAs, and potential xenomiRs were detected.

A stringent stepwise pipeline was designed to enable an accurate assessment of the presence of the potential xenomiRs after the removal of as many endogenous sequences, related to the host, or possible contaminants (Figure 1). A total of 1,660,099,441 reads related to 133 miRNA-Seq samples (from seven GSE IDs) were analyzed. After trimming the raw data, a total of 1,299,402,162 clean reads were obtained. The average sample sizes were 12.5 and 11 million reads before and after trimming, respectively. The summary of all the samples is provided in Supplementary Table S1.

### xenomiR detection in various datasets

#### Study number one (GSE136806)

**FIGURE 2:**
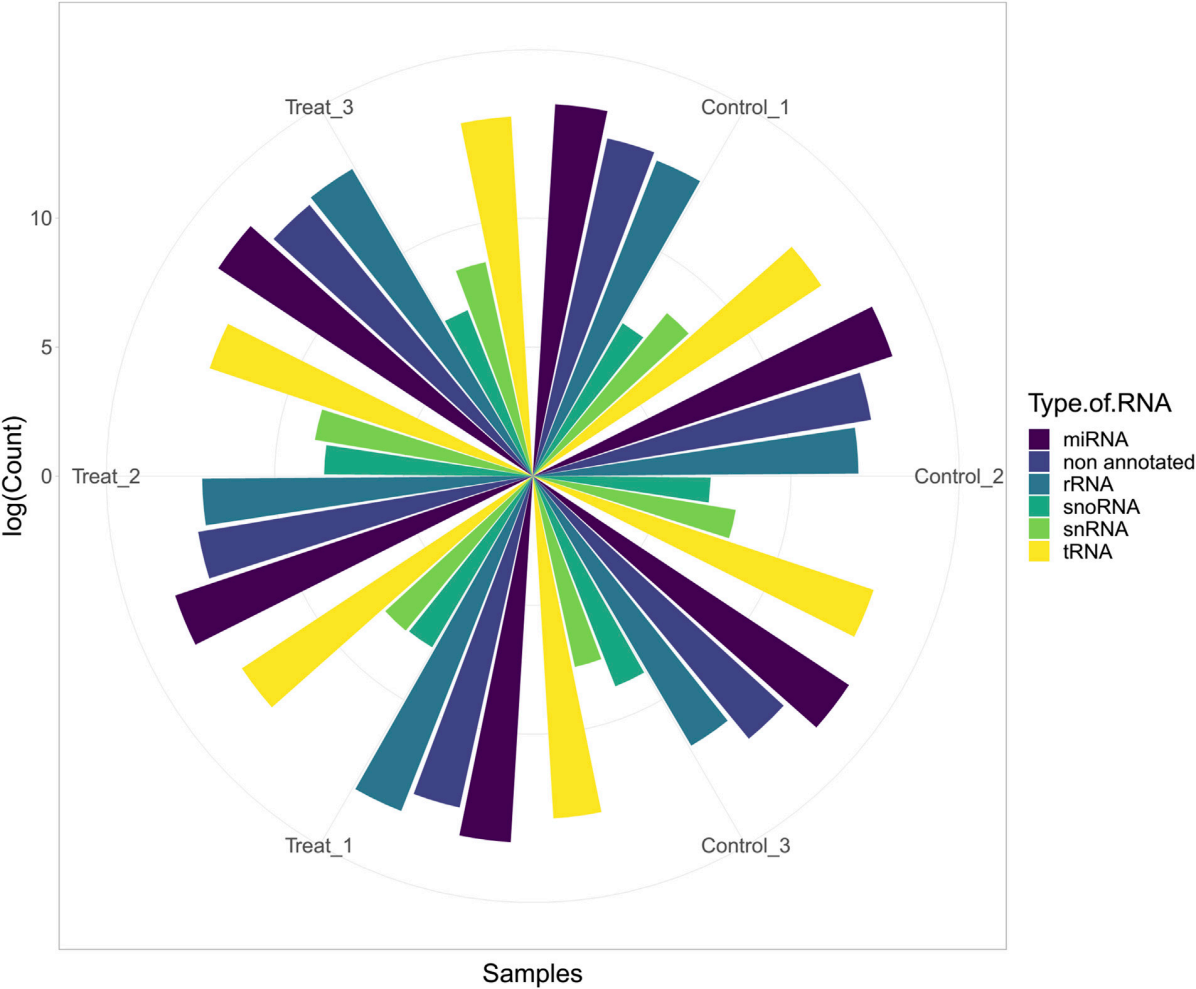
Abundance of host miRNA, tRNA, rRNA, snRNA, snoRNA, and non-annotated reads in each sample of study number one (GSE136806).

In this study, six cows were fed a normal diet (three cows) and a mixture of cotton seeds and soybean meal (three cows) (more details can be found in Supplementary Table S1). After initial processing, trimming, and filtering of the data based on the pipeline, 3,608,024 reads (601,337 reads per sample, on average) were retained for further analysis (Figure 2). Details of reading numbers related to different genes and non-annotated reads per sample are listed in Supplementary Table S3. To exclude the reads related to non-annotated genes of the bovine genome, the remaining reads were mapped to the bovine genome, and unmapped reads were extracted for aligning against the diet-derived miRNAs. A total of 897,697 reads (149,616 reads per sample, on average) were unmapped. Details of the percentage of mapping and number of unmapped reads per sample are provided in Supplementary Table S4.

Due to the fact that the cows were treated with cotton and soybean, miRNAs of these plants were considered as potential diet-derived miRNAs, and the unmapped reads were searched against them. Out of 897,697 reads, 32,726 reads were aligned, which belonged to 348 xenomiRs (Supplementary Table S5). The statistical analysis led to the identification of one differentially expressed miRNA, miR164, which was upregulated in the treated group (Supplementary Table S6). However, this miRNA was expressed in the control group as well. It is worth noting that the abundance of most miRNAs was low and in a small minority of samples. Also, many of the identified miRNAs were identical orthologs or paralogs (Supplementary Table S5). A xenomiR can be considered as potential diet-derived miRNA if it can only be expressed in all treated samples. Here, no xenomiR was found to be expressed in all treated samples, while that is not expressed in the control group. Comparing xenomiR abundance between control and treated samples (Supplementary Table S7), it is evident that the average abundance of the xenomiRs in both groups was similar. These findings suggest that the mapped reads to diet-derived miRNAs may not result from food ingestion, and it has a high probability of being contaminated during the preparation of samples in the laboratory or from the sequencing process in the sequencing center. The mean expression of miRNAs in cows treated with cotton and soy was 26.855, and in the control group, it was 59.638 (Supplementary Table S8).

#### Study number two (GSE117441)

In order to investigate the molecular regulatory mechanisms of milk protein production in dairy cows, three different diet groups (alfalfa hay, corn stover, and rice straw-based diets) were used in study number two. In this regard, the expression profile of miRNAs was assessed in five tissues (including the rumen epithelium, duodenum epithelium, jejunum epithelium, liver, and mammary gland). This study makes an opportunity to investigate the xenomiR hypothesis as the used diets are similar and only differ in one ingredient. The details of the diets are presented in Supplementary Table S1. Also, five tissues were investigated, and six experimental units (bovine) were assessed per diet. Based on the pipeline, the raw reads were trimmed and annotated (Supplementary Tables S3, S9). Non-annotated reads were aligned against the bovine genome, and 7,315,691 unmapped reads (1,527,667 reads from the rumen epithelium tissue, 1,799,780 reads from duodenum epithelium tissue, 1,228,401 reads from jejunum epithelium tissue, 1,012,718 reads from liver tissue, and 1,747,124 reads from mammary gland tissue) were remained to align against the diet-derived miRNAs (Supplementary Table S4).

Totally, 11,546 reads of 90 samples were aligned against the diet-derived miRNAs, covering 1,431 different plant miRNA sequences, and their abundances in each sample were obtained as presented in Supplementary Table S5. On average, the expression of each miRNA in duodenum epithelium tissue was 2.29 (alfalfa), 2.27 (rice straw), and 2.32 (corn); in jejunum epithelium tissue was 2.67 (alfalfa), 3.61 (rice straw), and 2.99 (corn); in rumen epithelium tissue was 1.63 (alfalfa), 1.52 (rice straw), and 1.41 (corn); in liver tissue was 2.47 (alfalfa), 1.96 (rice straw), and 1.83 (corn); and in mammary gland tissue was 2.38 (alfalfa), 3.09 (rice straw), and 1.64 (corn) (Supplementary Tables S7, S8). After statistical analysis, no miRNA was found to be differentially expressed between different diets (Supplementary Tables S6). Our findings revealed that no exogenous miRNA existed in all the tissues, comparing the different diets (each diet was used as a negative control for the other diets).

#### Study number three (GSE81616)

In order to investigate the presence of dietary-derived miRNAs in cows fed sunflowers, GSE81616 data were analyzed based on the pipeline, and detailed results are provided in Supplementary Tables S1, S3, S4, S9. Totally, 48,639 reads were retained to check for the presence of xenomiR. After aligning the remaining reads (48,638 reads) with the candidate miRNAs related to diet, 176 reads were aligned (Supplementary Table S5), which were related to 54 plant miRNAs. The mean expression of the miRNAs in cows fed sunflower oil was 1.904 and in cows in the control group was 2.269 (Supplementary Tables S7, S8). After statistical analysis, no miRNAs were found to be significantly differentially expressed (Supplementary Table S6).

#### Study number four (GSE113598)

In this study, two groups (including three mice per group) were fed a normal and a soy-enriched diet (normal diet plus soy) to elucidate the soy-induced liver miRNA changes in young male mice (Supplementary Table S1). The results of trimming, removing miRNAs of mice and miRNAs of close species to mice, and mapping against the mice genome are presented in Supplementary Tables S3, S4, S9, respectively. Finally, 1,329,009 reads were mapped against diet-derived miRNAs to assess whether soybean miRNAs are presented. Only 39 reads were aligned (Supplementary Table S5), which belonged to 11 xenomiRs. The mean expression of the miRNAs in mice fed soybean was 2.666 and in the control group was 1.666 (Supplementary Tables S7, S8). No miRNAs were found as differentially expressed genes (Supplementary Table S6).

#### Study number five (GSE81619)

miRNA expression profiles in the hypothalamus tissue of sheep fed a normal alfalfa diet (1.5 kg for each sheep) were compared with sheep fed alfalfa plus concentrate, in study number five (Supplementary Table S1). Our stringent pipeline was applied to get rid of the endogenous microRNAs (originated from sheep) (Supplementary Tables S3, S4, S9). Of 331,488 remaining reads, 314 reads related to 177 miRNAs were identified (Supplementary Table S5). The mean expression of the miRNAs in the treated and control groups were 1.801 and 1.277, respectively (Supplementary Tables S7, S8). After statistical analysis, no miRNA was found significant (Supplementary Table S6).

#### Study number six (GSE61025)

To investigate whether xenomiRs accumulate in mammary glands of goats, study number six was assessed. In this study, 10 goats were fed a hay-based diet. For 48 h before slaughtering, four goats continue consuming the diet *ad libitum* (control), and the others were food-deprived [FD (Supplementary Table S1)]. The data were subjected to our filtering pipeline, and the processed data (7,273,435 reads) were then used to assess the presence and abundance of xenomiRs (Supplementary Tables S3, S4, S9). Of these, 1,419 reads were aligned, which were related to 147 diet-derived miRNAs (Supplementary Table S5). The mean expression of miRNAs in food-deprived goats was 7.691 and in the control group was 5.993 (Supplementary Tables S7, S8). No differentially expressed miRNAs were identified after statistical analysis (Supplementary Table S6).

#### Study number seven (GSE92897)

To access the xenomiR hypothesis in study number seven, two experiments were conducted based on two species, including rat and pig (Table 1; Supplementary Table S1). In the rat experiment, 6,985,763 reads were retained after using our filtering pipeline (Supplementary Tables S3, S4, S9). Finally, 213 reads were aligned to 104 plant-derived miRNAs (Supplementary Table S5). The mean expression of plant-derived miRNAs in the control group and the rats treated with rice and potato was 1.46, 1.27, and 1.70, respectively (Supplementary Table S8). In the pig experiment, 2,297,927 reads were retained to check for the presence of xenomiRs (Supplementary Tables S3, S4, S9). Of these, 1,985 reads were aligned to 234 plant-derived miRNAs (Supplementary Table S5). The mean expression of the miRNAs in pigs fed corn and milk were 2.753 and 4.64, respectively (Supplementary Tables S7, S8).

**FIGURE 3:**
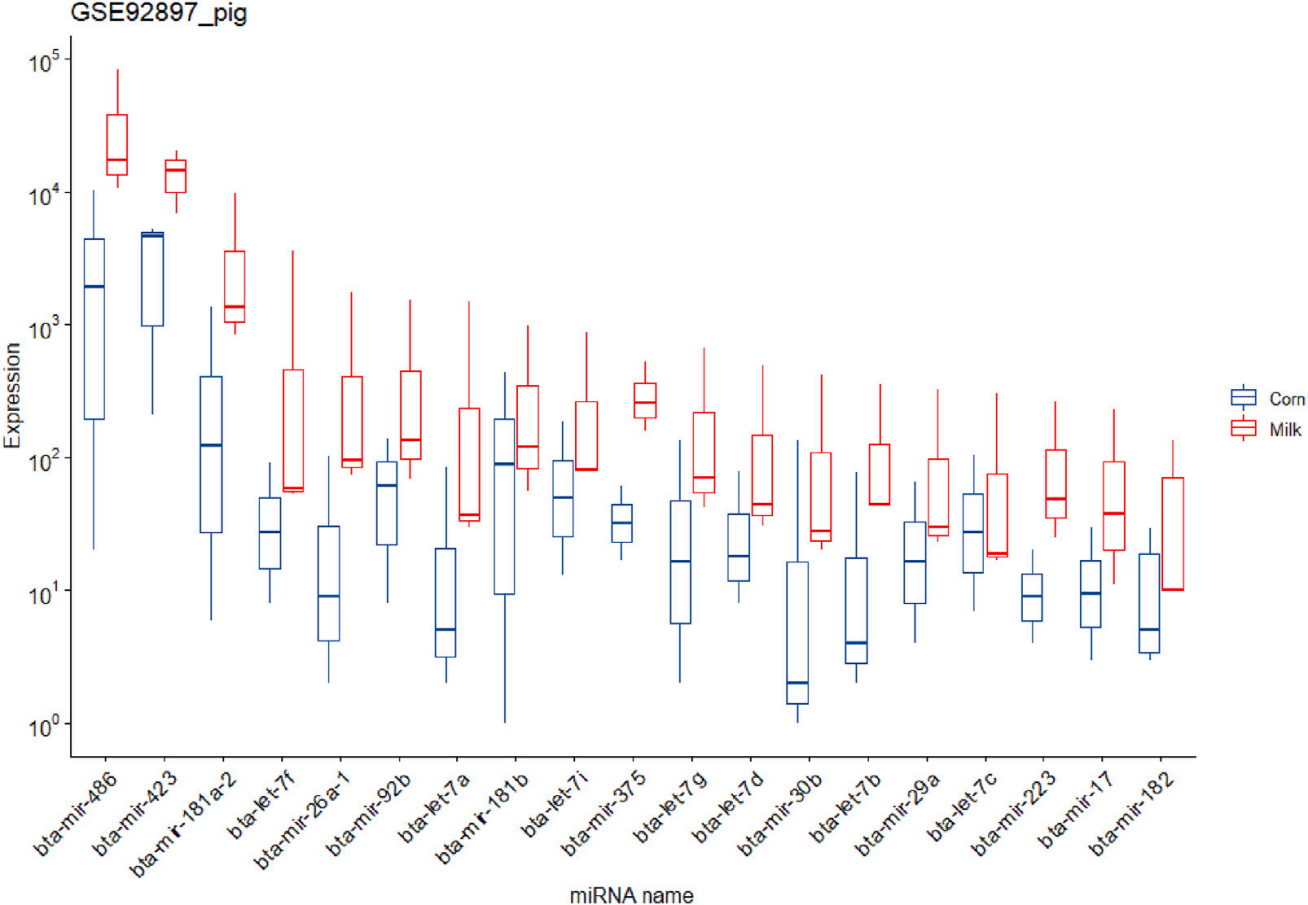
Expression of the 19 potential exogenous miRNAs that were predicted in the piglets fed corn and cow milk in study number seven.

It is worth noting that due to the fact that in the pig experiment a group was fed milk, the reads related to cows’ miRNAs can be removed in the filtering pipeline (in the step of removing all known species-related miRNAs). Taking into account this point, these data were analyzed again without this step and led to 56,064,228 reads for aligning against diet-derived miRNAs. In this regard, 684,590 reads were aligned against 538 diet-derived miRNAs (Supplementary Table S5). The mean expression of the miRNAs in pigs fed corn and milk was 165.78 and 526.05, respectively (Supplementary Tables S7, S8). Similar to the previous studies no significant differentially expressed miRNAs were found in both experiments (rat and pig method 1) (Supplementary Table S6). But according to the results of the second method of analyzing pig data, several significant differentially expressed miRNAs and overexpressed miRNAs were identified in the piglets that were fed cow milk (Supplementary Table S6), which is notable and controversial. Figure 3 shows the 19 identified overexpressed miRNAs in this study.

### Common xenomiRs among the different species and tissues

**FIGURE 4:**
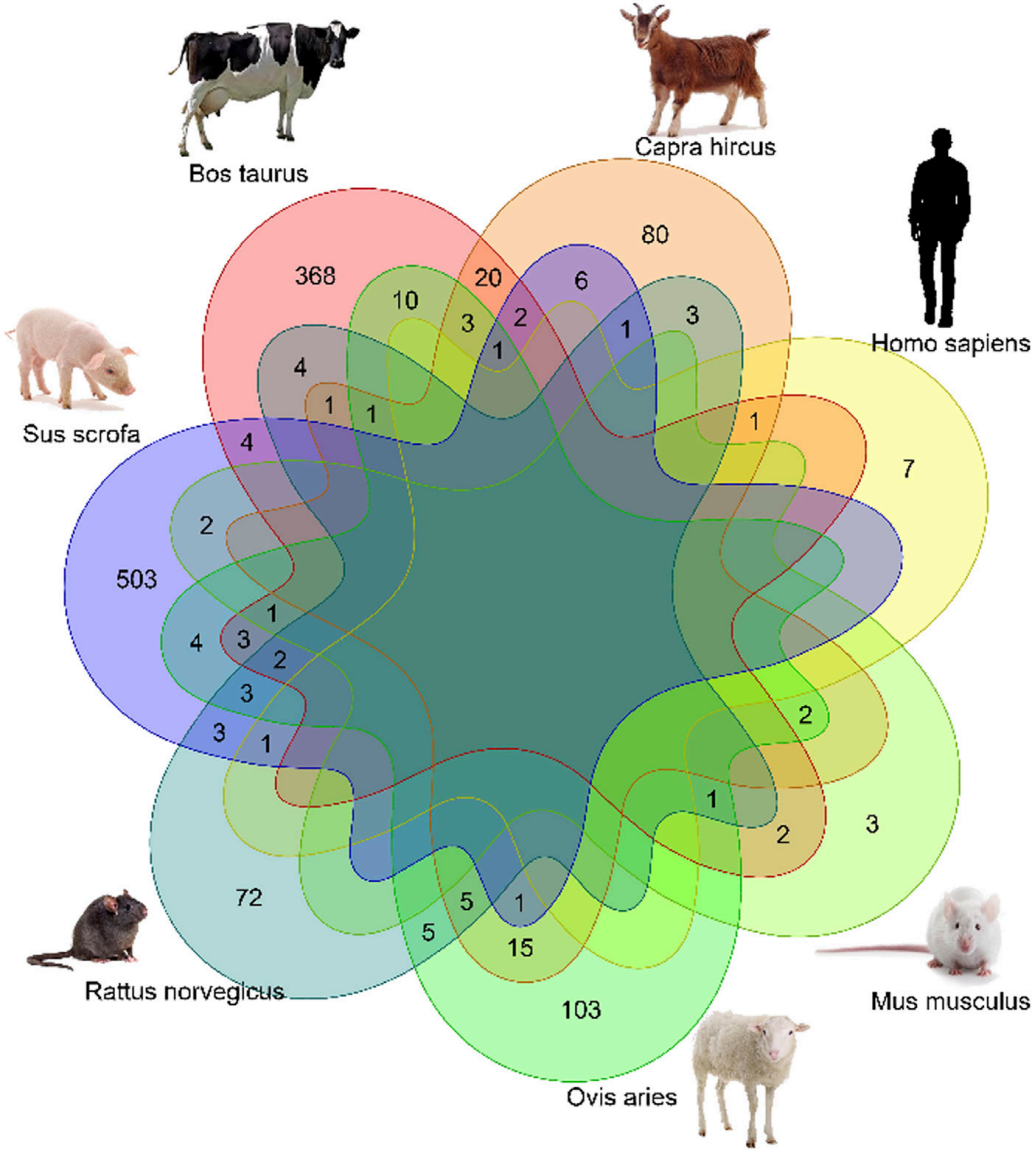
Venn diagram of miRNAs among the different species that were analyzed in this study.

**FIGURE 5:**
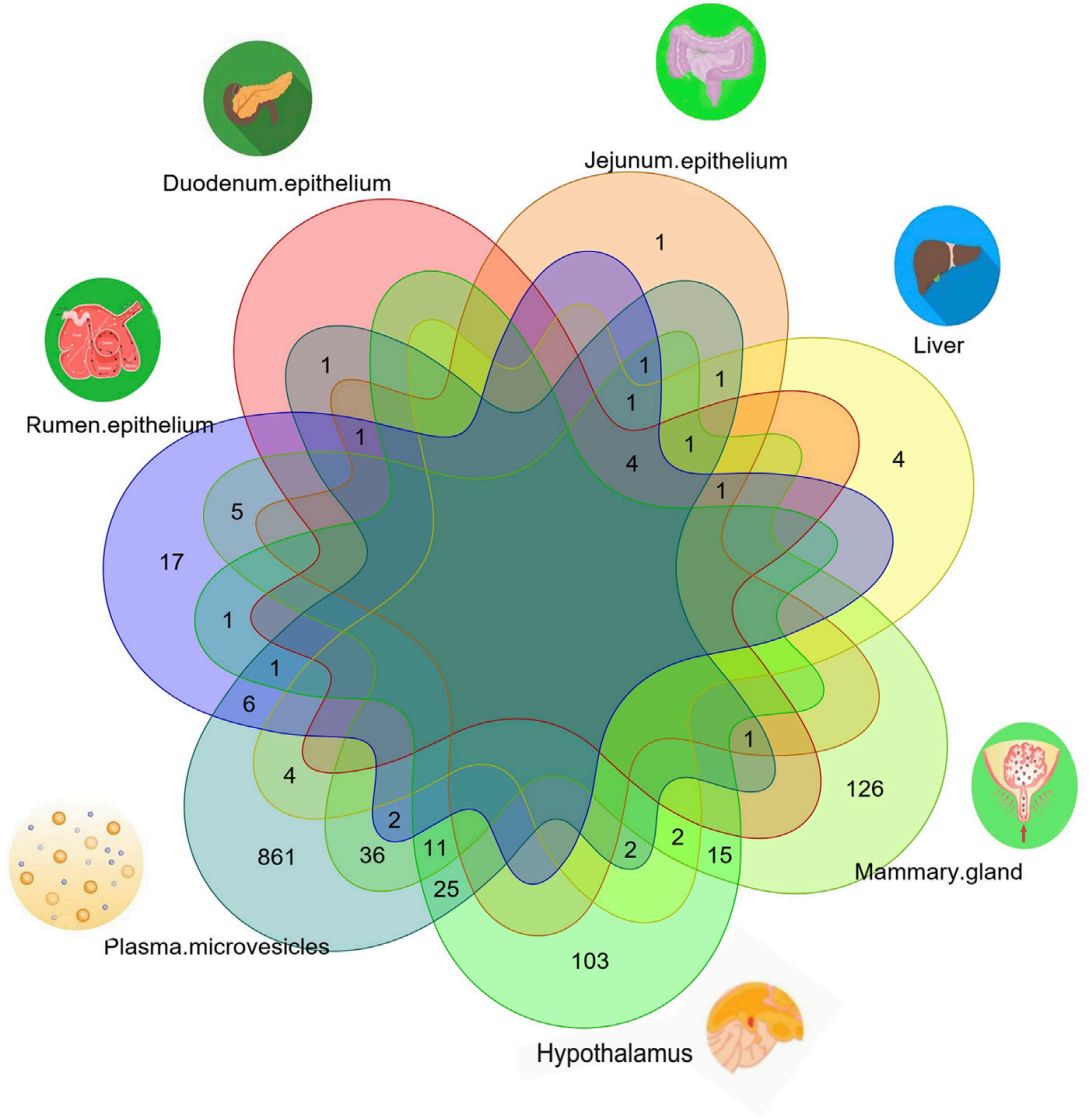
Venn diagram of miRNAs among the different tissues that were analyzed in this study.

**FIGURE 6:**
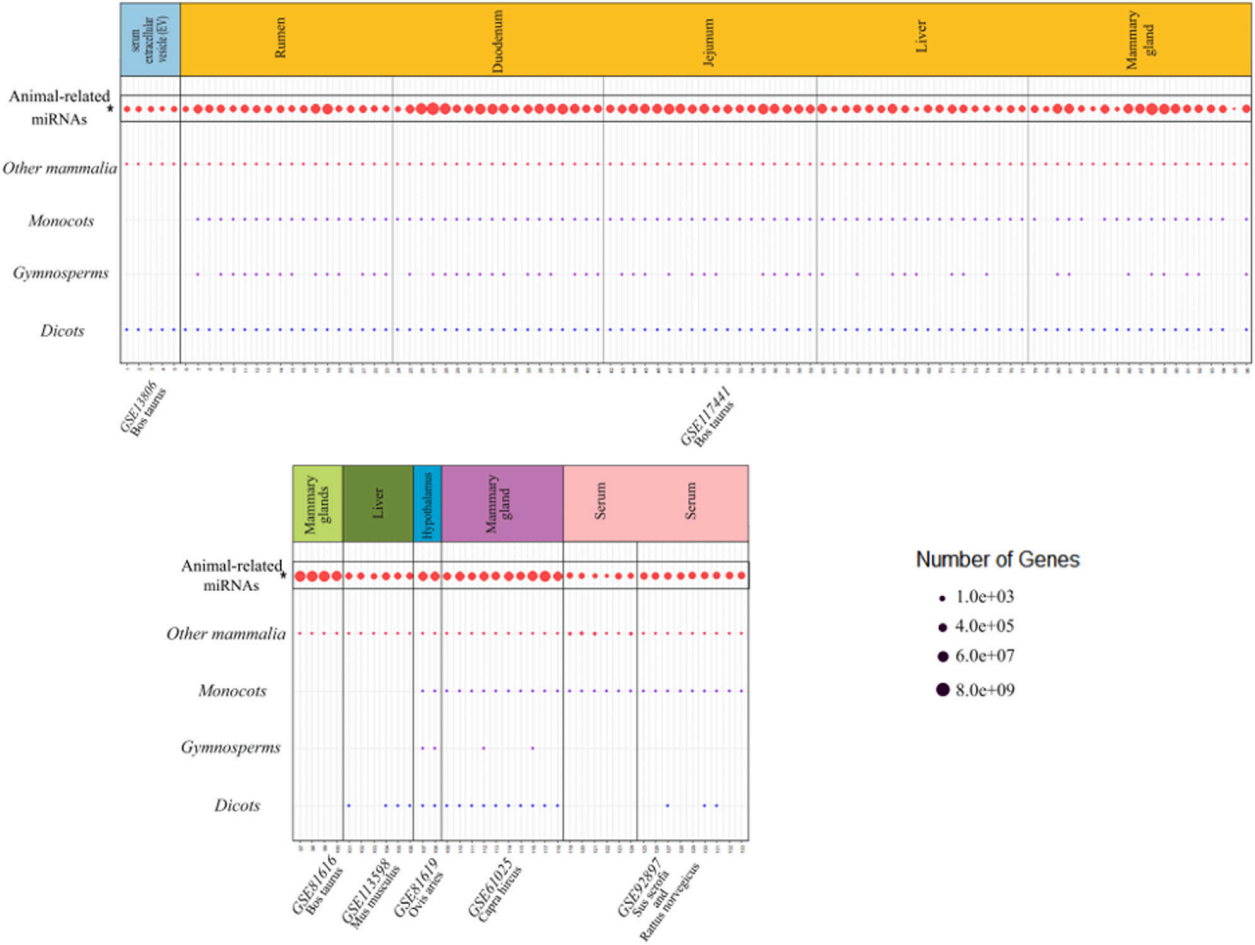
Comparison of the expression level of the species-related miRNAs with the potential diet-derived miRNAs in all the investigated samples in the present study. Every column represents one dataset. Samples are grouped in studies (facets) based on the database accession number (GSE number shown below each study). Every row represents one type of animal or plant clade. A dot indicates the presence of one or more xenomiRs belonging to the clade in the given sample. The dot size indicates how many distinct xenomiRs belonging to the clade were detected. XenomiRs are overall rarely present and lowly expressed compared to the species-related miRNAs. *The expressed miRNAs related to the animals that consumed diets of interest.

To investigate whether there were any common or unique xenomiRs among the species/tissues, a Venn diagram was applied using the “Venn” package of R software (Schwenk, 1984) (Figures 4, 5; Supplementary Table S10). This analysis was conducted based on the presence of miRNAs in each species/tissue, regardless of their relative abundance. The results showed that most of the potential diet-derived miRNAs were unique in each species or tissue. The highest number of common miRNAs among the species was found between *Bos taurus* and *Capra hircus* (20 miRNAs). On the other hand, mammary gland and plasma microvesicles included the highest number of common miRNAs, among the investigated tissues (36 miRNAs). Here, to compare the expression level of the species-related miRNAs to potential diet-derived miRNAs, the expression of miRNAs in all samples and in different tissues (including serum, liver, and mammary glands) was assessed. This analysis indicated that the species-related miRNAs (endogenous) and potential diet-derived miRNAs were presented in all tissues and body fluid samples (Figure 6). However, the species-related miRNAs were overall more abundant in the samples than the potential diet-derived miRNAs (xenomiR) (median of species-related miRNAs count: 360; potential diet-derived miRNAs count: 2.15).

## Discussion

Our daily diet not only provides the essential nutrients needed for survival and growth but also provides biologically active compounds to promote health and prevent disease (Liu, 2003). Continuous epidemiological studies have shown that regular consumption of plant foods such as fruits, vegetables, and whole grains are beneficial for metabolic disease, cancer, and age-related functional decline (Willett, 2002; Liu, 2004). Recent studies have shown that not only the miRNAs themselves but also the miRNAs in the food we eat may regulate the body’s cells after being absorbed through the gastrointestinal tract. This fascinating phenomenon suggests that miRNAs in the diet could act as a new class of biologically active agents in connection with the regulation of mammalian biological systems. In recent years, many studies have been conducted to investigate whether the xenomiRs detected in the samples are contaminated during experiments or bona fide diet-derived microRNAs. Given that the mechanism of this phenomenon is not well understood, more studies are needed to evaluate and confirm the xenomiR hypothesis. Accordingly, in this study, a total of 133 public miRNA-Seq samples were collected, comprising a total of 1,660,099,441 reads, to investigate the presence and abundance of xenomiRs. The datasets included 21 serum and 112 tissue samples; of these, 15 samples have previously been used to assess the xenomiR hypothesis, and 118 samples were applied for the first time in the present study to investigate whether xenomiRs accumulate in mammalian blood and tissues. All datasets were processed using a stringent computational workflow to ensure the results of different studies are comparable, and the detected miRNAs in the samples are of diet origin.

Since the xenomiR hypothesis is currently at an early stage, controversy exists regarding the detection and effectiveness of the reported diet-derived miRNAs and the repeatability of the published results. The results of this study did not support the xenomiR hypothesis, and our findings are in agreement with those of previous studies that reported that the identified xenomiRs probably originate from technical artifacts rather than dietary origin. It is interesting to note that our results suggest that vesicles in milk and blood contained more xenomiRs than tissues. However, a deeper look at the results showed that the identified xenomiRs are likely to be contaminants, and no evidence was found to support the cross-kingdom transfer of diet-derived miRNA hypothesis. In this regard, five reasons can be proposed:

1) The abundance of the identified xenomiRs was not high enough in the investigated tissues, except in body fluid vesicles (2.13 in tissues and 11 in body fluid vesicles). Also, it should be noted that these counts are substantially less than the total number of reads that are aligned against the animal miRNAs. The relative abundance of endogenous miRNAs is 62.97%, and the relative abundance of xenomiR is 0.0038% (Figure 6; Supplementary Tables S8, S11).
2) Most of the identified xenomiRs originate from a variety of sources (such as rodents, insects, and other plant species), from which the experimental units (animals) did not feed on. In this regard, we re-analyzed the study data of Liu et al. (Yuan et al., 2016; Ninomiya, Kawano, Abe, Ishikawa, Takahashi, Tamura, et al.) and found that many of the microRNAs belong to species from which the experimental units (in this study, humans) may not have fed (e.g., *Dinoponera quadriceps*, *Nematostella vectensis*, *Apis mellifera*, etc.) and are likely to be artificial/contaminated (Supplementary Table S12).
3) There is no consensus on biological replicate integration to consider candidate miRNAs as xenomiR. In other words, the animals that had been treated with the same diet showed different expression profiles of the candidate miRNAs as xenomiRs.
4) No common xenomiR was found among species or tissues. The top eight plant miRNA families with the highest xenomiR have been reported, with more than half of the xenomiRs belonging to these eight miRNA families (Zhao et al., 2018). Therefore, it is expected that in this study, common xenomiRs will be identified among different species or tissues. For this purpose, a van diagram was drawn to identify these xenomiRs (Figures 4, 5; Supplemental Tables S10), and no common xenomiRs were identified.
5) In addition, no evidence of transmission of dietary xenomiRs was found in controlled study No. 7, in which the authors examined the hypothesis of the presence or absence of xenomiRs. These findings indicate that the hypothesis of transfer of diet-derived miRNAs to the animal body is unexpected or can be occurred under special situations, as discussed as follows.

A number of functional miRNAs in cells/organs is one of the most important factors affecting their performance to have biologically significant effects. In our study, xenomiRs were found in some samples, but the expression was very low, about five (4.4738) reads per sample (Supplementary Table S8), which is negligible compared to endogenous miRNAs, about 50,000 counts per cell (Lappalainen et al., 2013). In this context, it is estimated that about 1000 to 10,000 copies of each miRNA are needed per cell to regulate its targets, which also depends on the strength of miRNA binding and the prevalence of the targets (Denzler et al., 2014). It is demonstrated that the miRNAs with low expression (<100 copies per cell) do not suppress their targets (Brown et al., 2007). Therefore, even if the identified xenomiRs are of dietary origin, their abundance is unlikely to be sufficient to affect cell transcripts. Briefly, xenomiRs were absent from most tissue samples, and their expressions were very low in the samples where they were present. All these evidence indicate that the absorption of diet-derived miRNAs into mammalian tissues following oral intake may not always be efficient. In this line, some of the reported xenomiRs are rejected when it comes to *in vivo* experiments (Philip et al., 2015; Witwer et al., 2016). However, further studies are required to determine the threshold concentration that diet-derived miRNAs need to reach to exert their biological effects in host organisms.

It is expected that diet-derived miRNAs be more abundant in some tissues that are higher exposed to dietary ingredients (such as the liver, rumen epithelium, duodenum epithelium, and jejunum epithelium) than the other tissues (such as mammary glands) (Kang et al., 2017). Study number two is an appropriate case to assess this hypothesis. Despite this, no difference was found between the tissues (Supplementary Table S5), and probably, the detected xenomiRs have a non-dietary origin (rumen epithelium = 1.52, duodenum epithelium = 2.29, jejunum epithelium = 3.09, liver = 2.09, and mammary_gland = 2.36).

Our findings are at odds with those of Liu et al.’s (Yuan et al., 2016; Ninomiya, Kawano, Abe, Ishikawa, Takahashi, Tamura, et al.), (Supplementary Table S12), which confirmed the hypothesis of translocation of dietary xenomiRs. It is important to note that the applied bioinformatic pipeline in Liu et al., study was not accurate. In their study, the clean reads were directly searched against the diet-derived miRNAs, without any filtering steps. Since sample contamination cannot be avoided in RNA sequencing experiments, using a stringent bioinformatics pipeline would engender higher confidence that the identified exogenous reads are not simply amplification or sequencing artifacts. Therefore, the results of Liu et al., study can be attributed to the point that they have not been applied to such a bioinformatics pipeline. In this regard, Witwer (2018) re-analyzed Liu et al., data and in agreement with the present study, failed to find any evidence to support the cross-kingdom transfer of exogenous miRNAs. The probable explanation for the reported diet-derived miRNAs in Liu et al., study is that the sequenced plant-derived miRNAs were artifactual/contaminations. For example, it is demonstrated that most of the reported xenomiRs in the previous studies on human originated from rodents, which is attributed to the proximity of these rodents to the laboratories (Kang et al., 2017). Hence, one of the most important filtering steps is searching the reads against these organisms. A comprehensive literature review indicates that the stringent bioinformatic pipeline and these filtering steps have not been applied in most of the studies in agreement with xenomiR hypothesis.

miRNAs packaged in vesicles in breast milk have been reported to play a regulatory role in the immune system of the infant consuming the milk (Admyre et al., 2007; Kosaka et al., 2010; Izumi et al., 2012; Munch et al., 2013). In this regard, this system has been reported as a new method of regulating the immune system in breast milk so that it can affect the function of the immune system in the infant. Some previous studies have also reported that colostrum miRNAs (early lactation) are more abundant than in mature milk (Gu et al., 2012; Sun et al., 2013; Xi et al., 2016). It is reported that the expression of immune-related miRNAs in colostrum is higher than in mature milk (Izumi et al., 2012; Na et al., 2015). In another study, the content of miRNAs from extracellular vesicles (EVs) purified from human and pig milk were examined and compared with published studies on EVs from human milk, cattle, goats, pigs, and pandas. Interestingly, several miRNAs were common among the species (e.g., members of the let-7 family, including let-7a, let-7b, let-7f, and miR-148a). In addition, these miRNAs have been reported to be involved in immune-related functions and regulating cell growth and message transmission (van Herwijnen et al., 2018). Here, to investigate the occurrence of this phenomenon in specific conditions and periods, study number seven was re-analyzed. In this experiment, the first group of newborn piglets was fed cow milk, and the second group of piglets was fed corn after weaning. As described in *Materials and methods*, two methods were applied to analyze these data. In the first method, to identify xenomiRs (cow’s and corn’s miRNAs), all miRNAs related to pigs were removed; then, the remaining data were aligned against the diet-derived miRNAs. This approach led to identifying no differentially expressed miRNAs (Supplementary Table S6), but there was a slight difference between the treatment and control groups (Supplementary Table S7). Since a group was fed cow milk, the reads related to cows’ miRNAs can be removed in the filtering steps. Hence, in the second method, the step of removing all known species-related miRNAs was denied. The results of this approach showed that the cow-derived miRNAs were expressed higher in animals fed cow milk than in the other group [such as mir-150 (Perri et al., 2018), mir-142, miR-223 (Perri et al., 2018), and the family of let-7s], which are probably originated from cow milk (Figure 3; Supplementary Table S5). On the other hand, some deeply conserved miRNAs like the let-7 family are present in all animals, and it is difficult to differentiate xenomiRs from animal-based miRNAs. Milk miRNAs are thought to be involved in several mechanisms of the immune system including differentiation and development of B and T cells, innate and adaptive responses (Alsaweed et al., 2015), and also neural development. Several miRNAs related to the infant’s immune system and neuronal development are presented in Supplementary Table S13. All microRNAs in Supplementary Table S13 were overexpressed in pigs fed cow milk. According to reports (Kosaka et al., 2010; Sun et al., 2013; Liao et al., 2017), higher expression of these miRNAs observed in this study probably originated from cow milk. In the first 6 months of life, it is possible that these miRNAs play a role in epigenetic regulation of an infant’s immune system development (Kosaka et al., 2010). Also, breastfed infants have been shown to exhibit a lower incidence of allergies, which may be related to the role of miRNAs in the development and prevention of autoimmune diseases (Alsaweed et al., 2015). The regulation of cell growth and differentiation by miRNAs is also considered to be a function of miRNAs in the infant. Several miRNAs found in milk, including miR-155 and miR-29a, are related to adipogenesis (Alsaweed et al., 2015). In addition, other milk miRNAs have been linked to the regulation of the central nervous system, as is the case with miR-118-2, whose target gene encodes a very abundant protein in the central nervous system (particularly in axons, so it is thought to be involved in intercellular communication). miRNAs have been shown to have neurocognitive benefits, perhaps acting as a regulator for neural development in infants. Nevertheless, the functions associated with the milk miRNAs discussed here are not fully demonstrated *in vivo* (Munch et al., 2013). Hence, further studies are needed to shed light on the underlying mechanisms regulating these processes.

Despite evidence from our results and several previous studies that rejected the xenomiR hypothesis (Witwer et al., 2013), there are many studies in favor of this hypothesis (Dickinson et al., 2013; Huang et al., 2018). Hence, the transmission ability of diet-derived miRNAs to host animals cannot be challenged only by contamination or other reasons such as undetectable abundance and irreproducible results. A comprehensive literature review showed that cross-kingdom communication can occur in different physiological and pathological conditions, as well as in certain organisms (Timmons et al., 2001) (such as honey bees (Gharehdaghi et al., 2021)). In this context, special diets or alterations in intestinal permeability have been reported to be effective in absorbing the miRNAs from the diet (Shi et al., 2012; Yang et al., 2015; Stephen et al., 2020), for example, many components of milk can enter and circulate in an infant’s body as they are born with low barrier integrity of their gut (Chernikova et al., 2018). Secretory immunoglobulins (IgA), leukocytes, lysozyme, lactoferrin, and other factors in breast milk contribute to passive immunity for infants and modulate their immune systems during development (Carlsson, 1994). In this regard, breast milk contains miRNAs with variable expressions during the developmental stage to provide nutritional requirements for the fetus (Benmoussa et al., 2016; Liao et al., 2017). It has been reported that the components inside the vesicles change the permeability of the intestinal. For example, overexpression of miR-122a induces the degradation of the occludin gene through binding to the 3'-UTR region. The degradation of this gene decreases occludin levels in enterocytes, thereby increasing the permeability of the intestinal tight junction (Ye et al., 2011). Interestingly, our results showed that expression of miR-122a was increased in piglets fed cow’s milk (study number seven).

Moreover, it is reported that absorption of diet-derived miRNAs in specific species of animals can be affected by the sequence of miRNAs (short length and high GC content could increase the stability of an RNA). The results of Yang et al. (2016) study showed that miRNAs with certain features are more stable in animal bodies. miRNA abundance in the consumed diet is one of the other important factors that affect the detectability of diet-derived miRNAs (Liang et al., 2015). Therefore, cross-kingdom regulation probably occurs in certain situations, which is an explanation for some studies, like the present study, that could not demonstrate the cross-species transfer of miRNAs.

## Conclusion

Overall, the results of the computational analyses performed in the present study showed that the potential diet-derived small RNAs that are detected in low frequency in animal samples are most likely due to contamination (false positive). There is also evidence to support the idea that pollution is actually the main cause of these findings. It is also in agreement with the theory of the transfer of small RNAs under certain conditions and periods, for example, the transfer *via* milk’s EV to infant cells. The exact implications of these findings and their importance in other biological processes require further research, both *in vitro* and *in vivo*.

## Data Availability

Publicly available datasets were analyzed in this study. The names of the repository/repositories and accession number(s) can be found in the article/Supplementary Material.
