# Supplementary material for: Investigation of the transability of dietary small non-coding RNAs to animals
Source: Front Genet. 2022 Aug 30;13:933709. doi: 10.3389/fgene.2022.933709 (PMC9483711; doi:10.3389/fgene.2022.933709)
Supplement: Supplementary file 3 [file Table1.DOCX]

**Supplementary File S1:**

Summary of the investigated studies in the present study

**Study number one (GSE136806)**

Summary of this study: In this study, the structure of small RNAs related to cow serum was studied and their effect on the immune system was investigated. In addition, it has been reported that different diets can affect the expression of small RNAs. The results of this study suggest that individuals can use effective diets to change the physiological state of animals. For this purpose, they substituted small amounts of cotton and soybeans for alfalfa in the diets of the tested cow. Blood EVs were isolated. The findings of this study led to the identification of 359 types of small RNAs in cattle. Analysis of the KEGG pathways showed that differently expressed small RNAs were associated with hormone messenger pathways and protein metabolism. In this study six cows were fed, normal diet (three cows) and mixture of cotton seeds and soybean skin (three cows) (table 1).

**Table 1.** The Total Mixed Ration (TMR) formula of the control group and the tested group. related to the study number one (GSE136806)

| **Items** | **Experiment Diet** | |
| --- | --- | --- |
|  | **Control Group** | **Tested Group** |
| Ingredient, % of DM |  |  |
| Alfalfa hay | 18.67 | 9.70 |
| Corn silage | 27.39 | 24.88 |
| Steam-flaked corn | 23.20 | 24.25 |
| Soybean meal | 8.22 | 8.21 |
| Cottonseed meal | 9.54 | 9.54 |
| Beet pulp | 5.68 | 5.68 |
| Distillers dried grains with solubles | 3.78 | 3.77 |
| Whole cotton seed | — | 5.72 |
| Soybean hull | — | 4.73 |
| Bergagat T300 | 1.04 | 1.04 |
| Premix | 1.86 | 1.84 |
| Chemical composition, % of DM |  |  |
| CP | 16.02 | 16.34 |
| EE | 4.20 | 5.18 |
| RDP(%CP) | 58.61 | 56.64 |
| NDF | 31.01 | 31.84 |
| f NDF | 20.92 | 15.67 |
| peNDF | 11.61 | 11.63 |
| ADF | 21.78 | 22.23 |
| NFC | 40.48 | 38.92 |
| Starch | 25.35 | 25.49 |
| NEL, Mcal/kg | 1.61 | 1.62 |

**Study number two (****GSE117441)**

In this study, researchers investigated the molecular regulatory mechanisms of milk protein production in dairy cows by studying the miRNAomes of five key metabolic tissues involved in protein synthesis and metabolism from dairy cows fed high- and low-quality diets. In total, 340, 338, 337, 330, and 328 miRNAs were expressed in the rumen-epithelium, duodenum-epithelium, jejunum-epithelium, liver, and mammary gland tissues, respectively. Some miRNAs were highly correlated with feed and nitrogen efficiency, with target genes involved in transportation and phosphorylation of amino acid (AA). Additionally, low-quality forage diets (corn stover and rice straw) influenced the expression of feed and nitrogen efficiency-associated miRNAs such as miR-99b in rumen-epithelium, miR-2336 in duodenum-epithelium, miR-652 in jejunum-epithelium, miR-1 in liver, and miR-181a in mammary gland. Ruminal miR-21-3p and liver miR-2285f were predicted to regulate AA transportation by targeting ATP1A2 and SLC7A8, respectively. Furthermore, bovine-specific miRNAs regulated the proliferation and morphology of rumen epithelium, as well as the metabolism of liver lipids and branched-chain AAs, revealing bovine-specific mechanisms. Our results suggest that miRNAs expressed in these five tissues play roles in regulating transportation of AA for downstream milk production, which is an important mechanism that may be associated with low milk protein under low quality forage feed. For this purpose, 18 cows were fed with three diets with different sources of forage including alfalfa (6 heads), rice straw (6 heads) and corn (6 heads) for 90 days. Details of the dietary composition of diets are given in Table 2.

**Table 2**. Ingredients of the 3 experimental diets based on alfalfa, corn stover, and rice straw, related to the study number two (GSE117441)

| **Ingredient, % of DM** | **Treatment** | | |
| --- | --- | --- | --- |
|  | **AH** | **CS** | **RS** |
| Ground corn grain | 27.0 | 27.0 | 27.0 |
| Wheat bran | 5.1 | 5.1 | 5.1 |
| Soybean meal | 12.7 | 12.7 | 12.7 |
| Cottonseed meal | 4.3 | 4.3 | 4.3 |
| Beet pulp | 1.0 | 0.0 | 0.0 |
| Corn silage | 15.0 | 15.0 | 15.0 |
| Alfalfa hay | 23.0 | 0.0 | 0.0 |
| Chinese wild grass hay | 7.0 | 0.0 | 0.0 |
| Corn stover | 0.0 | 30.0 | 0.0 |
| Rice straw | 0.0 | 0.0 | 30.0 |
| Urea | 0.0 | 1.0 | 1.0 |
| Premix[2](https://www.sciencedirect.com/science/article/pii/S0022030214006481#tblfn0010) | 4.9 | 4.9 | 4.9 |

**Study number three (****GSE81616)**

Summary of this study: The aim of this study was to identify small RNAs whose expression in the mammary gland of cattle is modulated using sunflower oil supplementation. Small RNAs were obtained from the mammary glands of lactating cows after receiving a diet containing 4% sunflower oil and control. The results of this study led to the identification of 272 small RNAs. The findings of this study showed that the significant decrease in expression of miR-142-5p and miR-20a-5p is due to the use of sunflower supplements. The target genes of these two different small RNAs were predicted. One of the predicted targets was the ELOVL6 gene, which is linked to fat metabolism. In this study, 11 healthy cows were fed two different diets (LF و LF-SO) (Table 3). Each feeding period was for 28 days. The effect of sunflower oil supplementation on the product and milk composition has been previously reported.

**Table 3**. Ingredient composition of the ingested experimental diets, related to the study number three (GSE81616)

| **Items** | **Treatment** | |
| --- | --- | --- |
|  | **LF** | **LF-SO** |
| Natural grassland hay | 45.9 | 50.7 |
| Corn grain | 42.7 | 29.5 |
| Dehydrated beet pulp | 2.9 | 3 |
| Soy bean meal | 2.8 | 6.8 |
| RS meal | 5.7 | 6 |
| SO | 0 | 4 |

**Study number four (GSE113598)**

Soy-based diets have triggered the interest of the research community due to their beneficial effects on a wide variety of pathologies like breast and prostate cancer, diabetes, and atherosclerosis. However, the molecular details underlying these effects are far from being completely understood and several recent attempts have been made to elucidate the soy-induced liver transcriptome changes in different animal models. Here researchers used Next Generation Sequencing to identify a set of two microRNAs specifically modulated by short-term soy-enriched diet in young male mice and estimate their impact on the liver transcriptome assessed by microarray. Clustering and topological community detection (CTCD) network analysis of STRING generated interactions of transcriptome data led to the identification of five topological communities of genes characteristically altered and putatively targeted by microRNAs upon soy diet intervention. In this study, two groups of three 12 weeks old male mice housed in Udel® polysulphone cages, on a 12-hour light‐dark cycle were fed ad libitum granulated regular chow (Cantacusino Institute, Bucharest) and granulated soy‐enriched chow (25% soy bean) for 28 days. On day 28, the animals were sacrificed and approximately 0.5 g of liver tissues have been collected, immediately, immersed in RNAlater stabilization solution (Qiagen) and stored at −80°C until its further use.

**Study number five (GSE81619)**

Summary of this study: Establishing estrus plays an important role in the sheep industry to improve meat and wool production. A normal diet can cause estrus and thus the end of the estrus season earlier than ewes who have been restricted in their diet. However, the physiological endocrine mechanisms that increase the prevalence of estrus are unclear. In the present study, differences in the profile of Hypothalamus RNAs in estrus ewes were investigated using Illumina HiSeq sequencing technology. The results showed that nutritional status plays an important role in regulating estrus in sheep. The PLA2G4D gene can directly regulate ovarian follicle growth or indirectly affect leptin secretion, which regulates the endocrine and physiological systems of the reproductive system during the infertility season. Healthy Kazakh ewes (36 heads) were randomly divided into two groups (18 heads in each group). The control group was fed with a normal alfalfa diet (1.5 kg for each sheep) and free access to water. The treatment group with better nutritional status including a concentrate supplement (0.3 kg for each sheep) was fed with higher nutrient content than the control diet.

**Study number six (GSE61025)**

Summary of this study: Nutrition affects the composition of milk, so it will also affect its nutritional properties. Nutrition also alters the expression of breast genes. The aim of this study was to describe RNAs whose expression is regulated by feeding in the mammary gland of lactating goats, as they may provide information to identify the mechanisms of biosynthesis and secretion of milk components. Twelve alpine goats were selected for late lactation based on homogeneity of milk yield, number of calves and genotype of α-S1-casein gene. The goats were fed an orchard grass hay-based diet with a 35:65 forage to concentrate ratio during a 2-wk preexperimental period. For 48 h before slaughtering, 6 goats consumed this diet ad libitum (control goats) and the 6 others were food deprived (FDd). Goats were milked at 0800 and 1600. Goats were housed in individual stalls, had free access to water, and were fed twice daily (except during the 48-h FD) just after milking. The goats were cared for and handled in compliance with the INRA Animal Care Committee guidelines.

**Study number seven (GSE92897)**

Summary of this study: In this study, two different animal experimental designs have been investigated. These experiments were performed in two different animal centers. In the first experiment, male Sprague-Dawley rats weighing 300 g were randomly divided into three groups (3 animals in each group). two groups were fed exclusively with potatoes or rice. As a control group, three rats in the third group were fed a diet without treatment. The second experiment was performed on three-breed guinea pigs (Dorox * Danish * Yorksier). Piglets were divided into two groups (3 pigs in each group). the first group was fed a diet based on cow's milk and the second group was fed a diet based on corn. Serum RNA extracted from two different animal models were both extracted according to the manufacturer's instructions and sequenced with the Illumina HiSeq2000 device. In this study, the researchers reported that no transfer of small plant RNAs or small bovine milk-specific RNAs into the blood of rats and piglets. In summary, the comprehensive computational and experimental results of these researchers show that xenomiRs are derived from contamination.
