# Supplementary material for: Investigation of the transability of dietary small non-coding RNAs to animals
Source: Front Genet. 2022 Aug 30;13:933709. doi: 10.3389/fgene.2022.933709 (PMC9483711; doi:10.3389/fgene.2022.933709)

**Supplementary File S7:**

Expression of the potential exogenous miRNAs with highest expression in samples of different investigated studies.

**Study number one (GSE136806)**


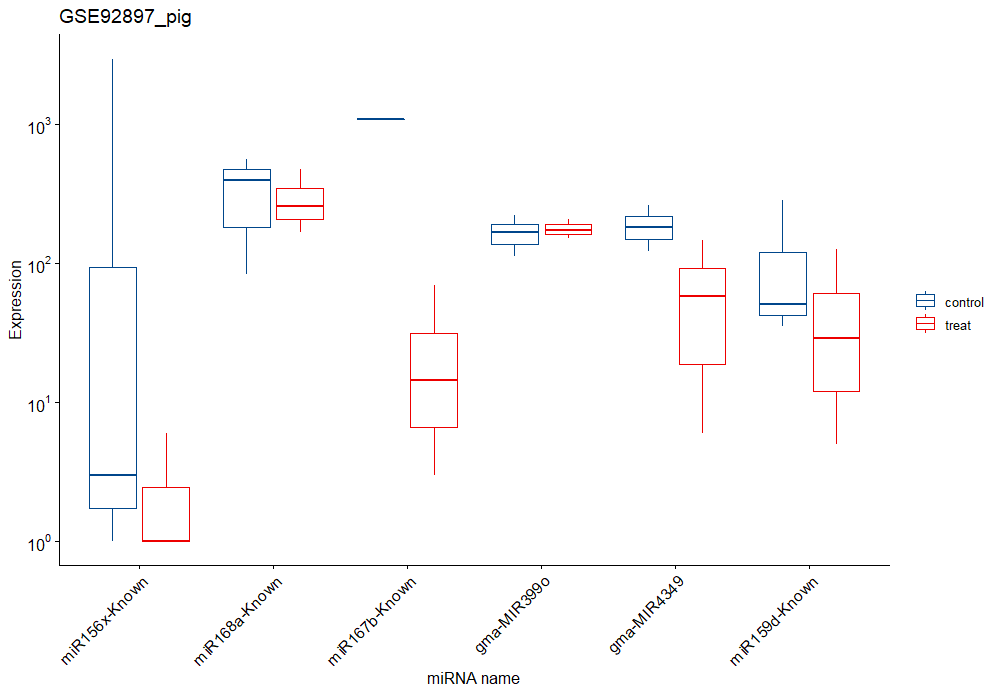


**Study number two (****GSE117441)**

1-Rumen-epithelium


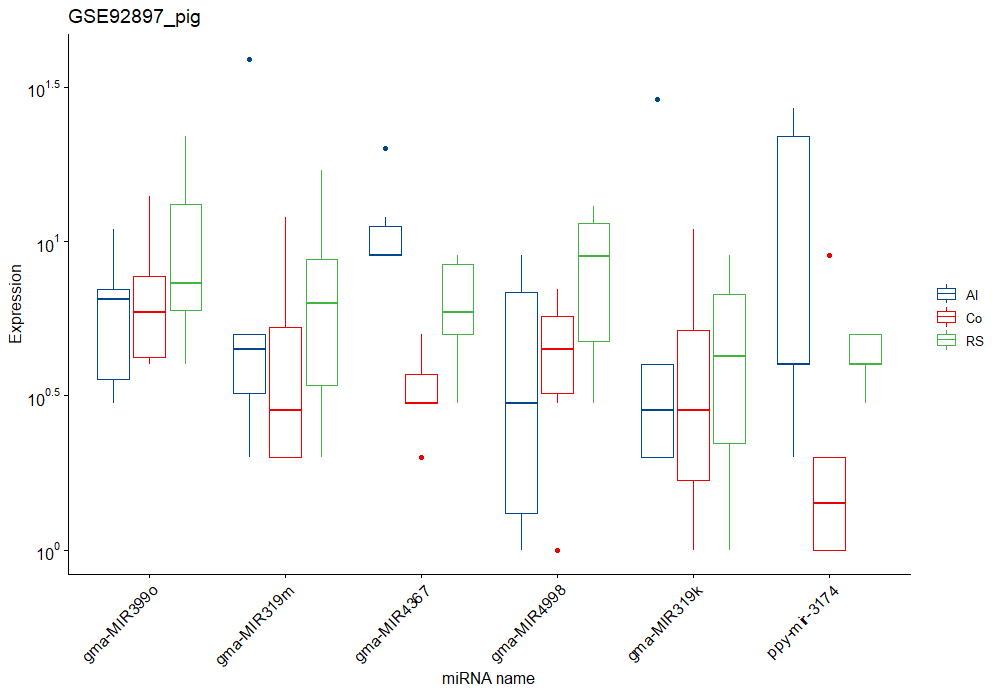


2-Duodenum-epithelium


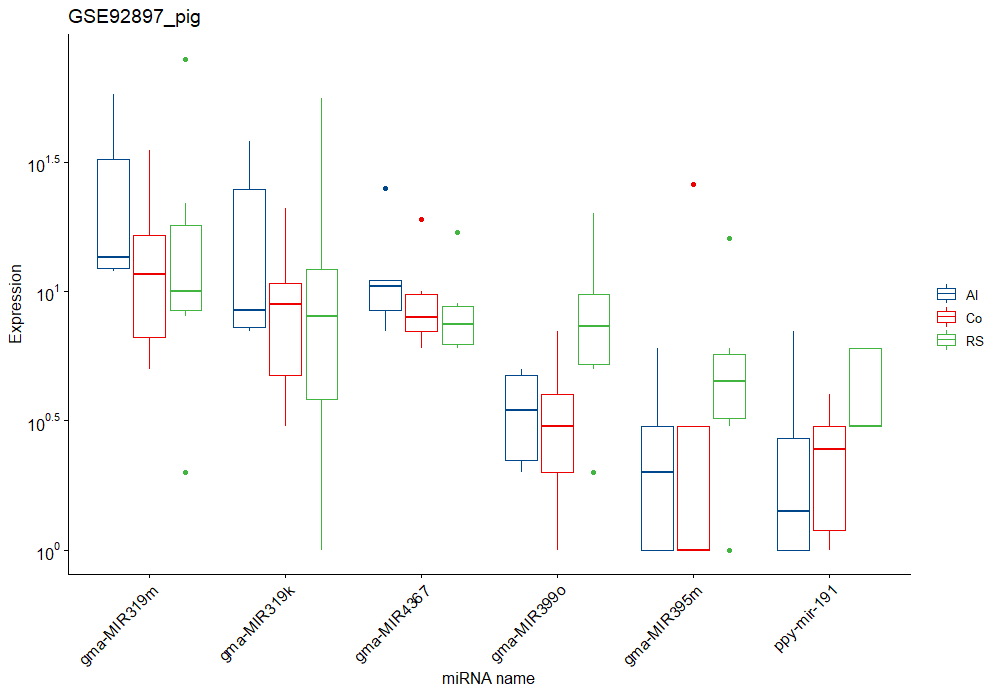


3- Jejunum-epithelium


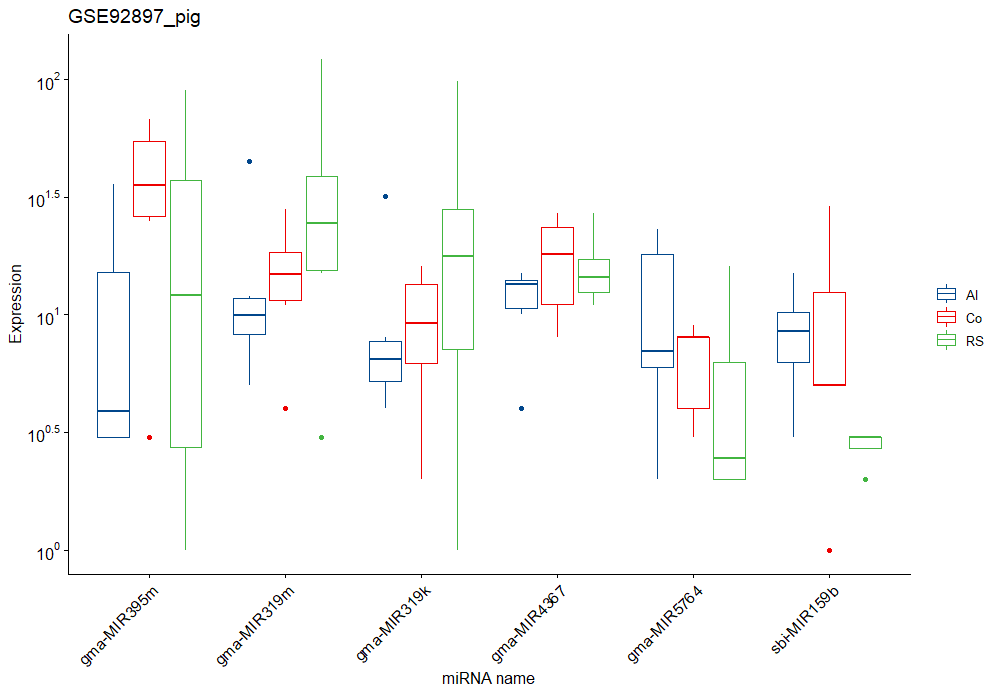


4- Liver


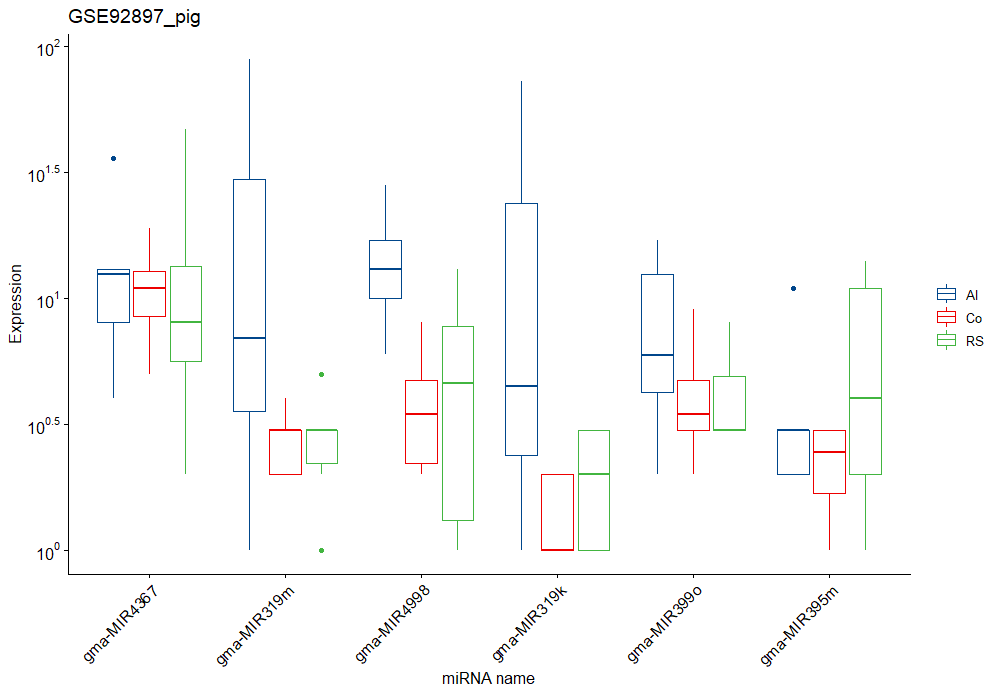


5- Mammary gland


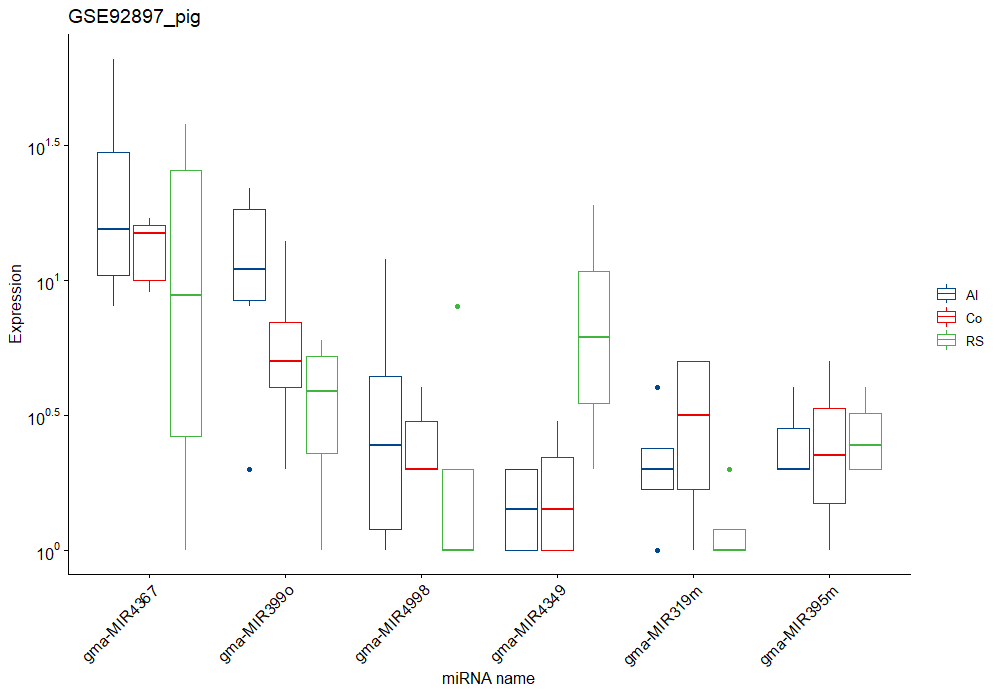


**Study number three (****GSE81616)**


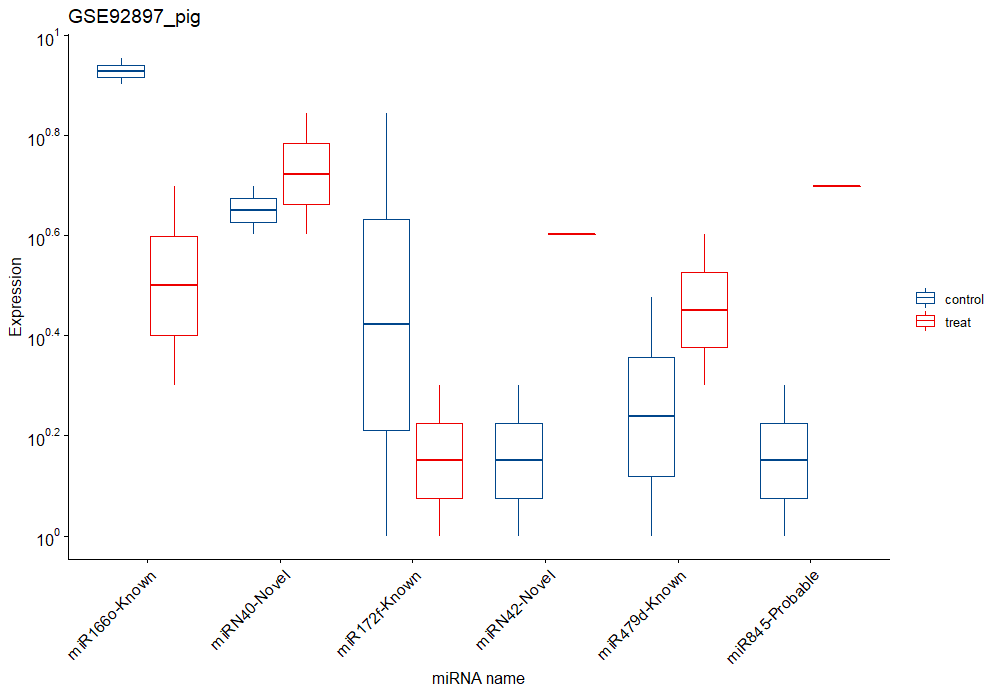


**Study number four (GSE113598)**


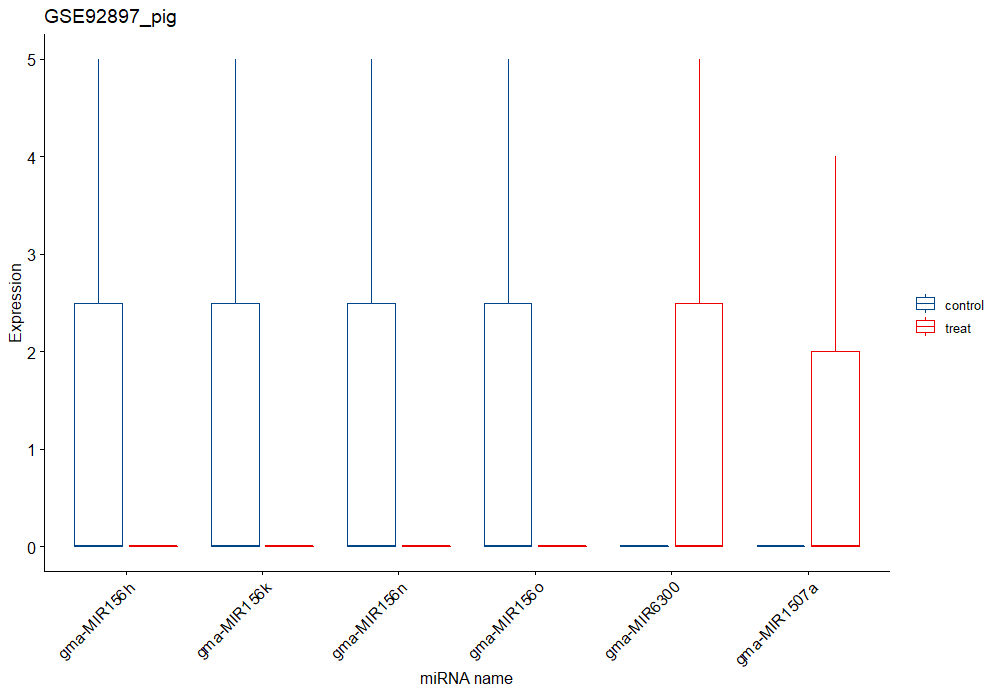


**Study number five (GSE81619)**


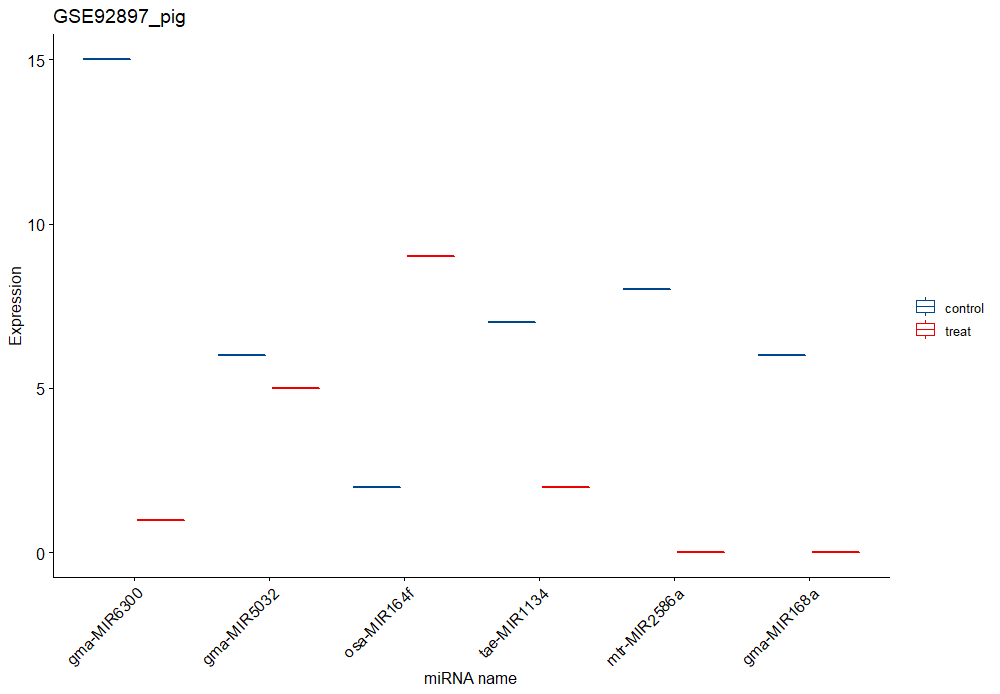


**Study number six (GSE61025)**


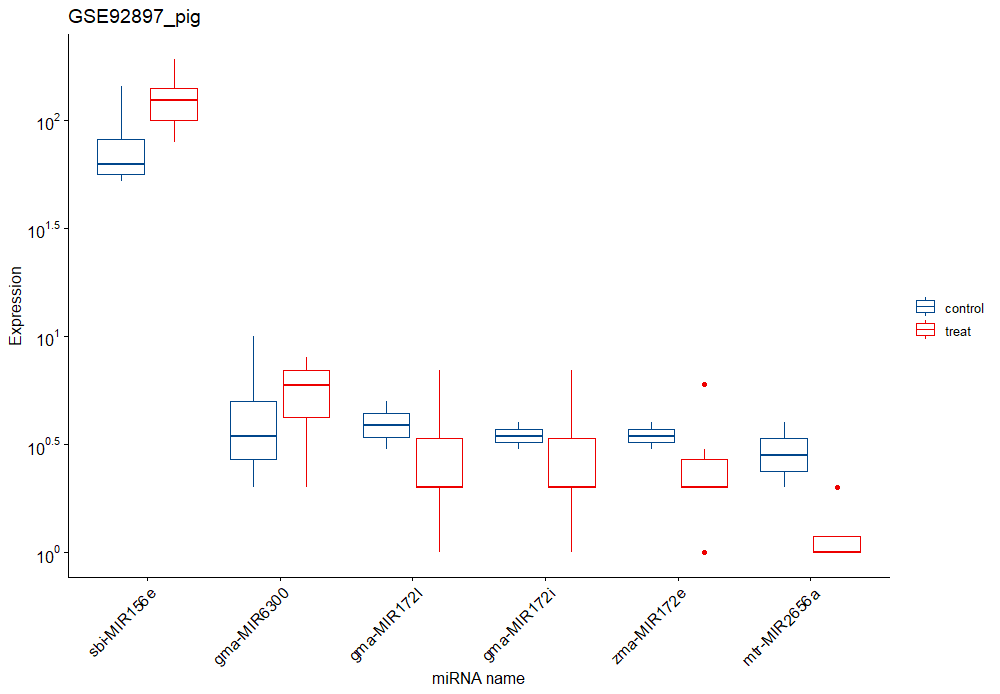


**Study number seven (GSE92897)**

rats’ experiment


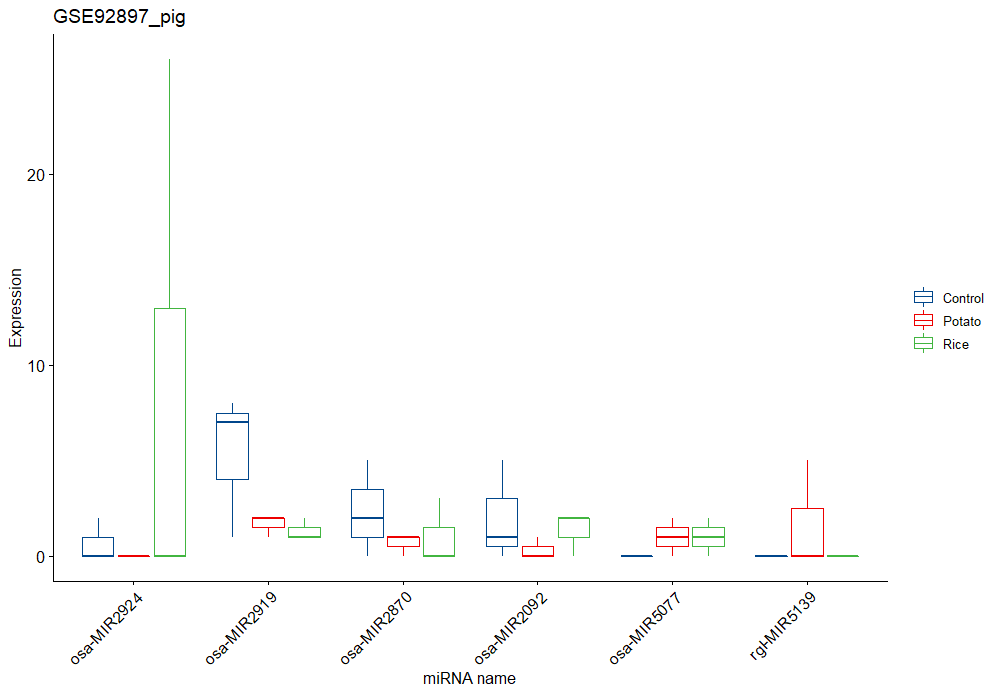


pigs’ experiment (method 1)


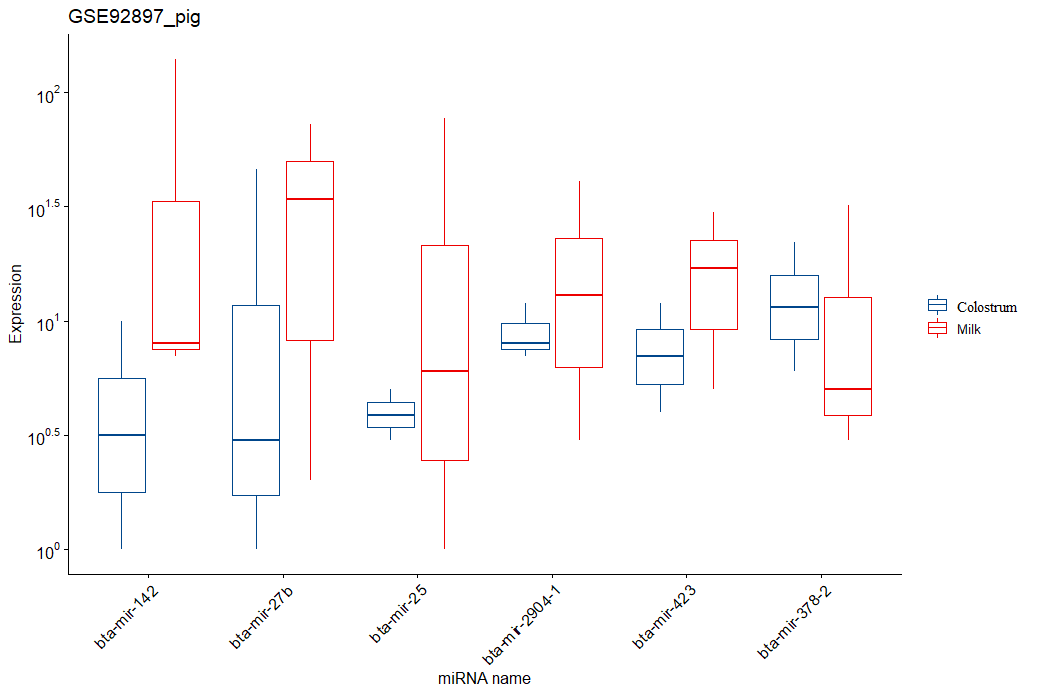


pigs’ experiment (method 2)


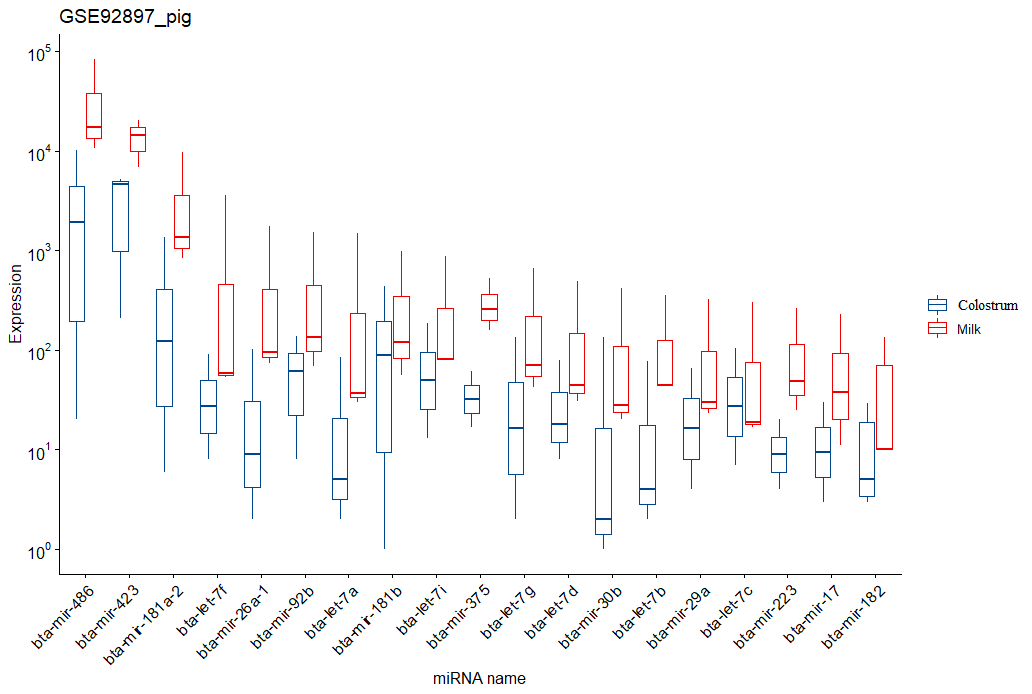

Supplement: Supplementary file 4 [file Table7.DOCX]
